# Effect of Aging on the Mechanical Properties of Highly Transparent Fluoropolymers for the Conservation of Archaeological Sites

**DOI:** 10.3390/polym14050912

**Published:** 2022-02-24

**Authors:** Maria Paola Bracciale, Lorena Capasso, Fabrizio Sarasini, Jacopo Tirillò, Maria Laura Santarelli

**Affiliations:** Department of Chemical Engineering Materials Environment (DICMA), University of Rome Sapienza, Via Eudossiana 18, 00184 Rome, Italy; lorena.capasso@gsk.com (L.C.); fabrizio.sarasini@uniroma1.it (F.S.); jacopo.tirillo@uniroma1.it (J.T.)

**Keywords:** fluorinated polymers, UV-A accelerated aging, mechanical behavior, archaeological conservation

## Abstract

In recent years, fluoropolymers have found numerous applications in the architectural field because of their combination of mechanical-chemical resistance and high transparency. In the present work, commercial fluorinated polymers, such as perfluoro alkoxy (PFA) and ethylene tetrafluoroethylene copolymer (ETFE), have been evaluated for use as protective and transparent layers on monumental and archaeological sites (to preserve mosaics or frescoes) during the phases of restoration or maintenance outdoors. Considering this specific application, the present study was developed by evaluating the evolution of the mechanical (tensile, tear propagation resistance, and low-velocity impact tests) and chemical (FTIR and DSC analysis) properties of the films after accelerated UV aging. The results that were obtained demonstrated the high resistance capacity of the ETFE, which exhibits considerably higher elastic modulus and critical tear energy values than PFA films (1075.38 MPa and 131.70 N/mm for ETFE; 625.48 MPa and 59.06 N/mm for PFA). After aging, the samples exhibited only a slight reduction of about 5% in the elastic modulus for both polymers and 10% in the critical tear energy values for PFA. Furthermore, the differences in impact resistance after aging were limited for both polymers; however, the ETFE film showed higher peak force than the PFA films (82.95 N and 42.22 N, respectively). The results obtained demonstrated the high resistance capacity of ETFE films, making them the most suitable candidate for the considered application.

## 1. Introduction

The conservation of archaeological sites has always sparked discussion among conservation scientists because of the need to solve several problems simultaneously. For example, an archaeological site requires constant and continuous maintenance, which includes restoration interventions, pipe adaptation works, or archaeological excavations. During these activities, however, it is necessary to cover and protect important archaeological artifacts such as mosaics, frescoes, or stuccos, thereby removing them from public view, with a significant economic impact on the archaeological site due to temporary closures.

In order to limit these problems, the maintenance schedule should be organized in concert with arranging visits to the artifacts, with the intention of combining their visibility with protection from environmental conditions, dust, falling objects, and restoration products (e.g., dripping from cement injections). For this reason, suitable materials need to be identified.

Generally, materials such as bentonite on non-woven textile, or neoprene or PVC mats are used for coverage, but they do not guarantee visibility or control of conservation during the restoration works.

In the present study, the use of ETFE (copolymer of tetrafluoroethylene and ethylene) films is suggested as protective coverage for ancient floors or painted walls. Indeed, considering their high transparency and high mechanical strength, they can provide adequate protection for the artifacts during the restoration and conservation phases.

Fluorinated polymers are characterized by their extraordinary properties and high performance, even in very severe conditions. The main polymer of this family is polytetrafluoroethylene (PTFE), a perfluorinated polymer comprising only carbon and fluorine atoms, more widely known as Teflon. In general, the higher the fluorine content, the stronger the properties deriving from the presence of this element [1,2].

Considering the low processability of PTFE, several new types of fluoropolymers, such as FEP (a copolymer of perfluoroethylene and perfluoropropylene) and PFA (perfluoroalkoxy), have been optimized. FEP is the second most significant fluoropolymer in terms of sales, but alongside its enormous advantage of extrusion processability, it has several disadvantages when compared with PTFE: the thermal stability is lower (about 200 °C, against 260 °C for tetrafluoroethylene); its tensile strength is 10–15% lower; and its solubility in solvents is higher [1]. PFA, introduced in 1973 and featuring a pendant group bonded to the main chain through an oxygen atom, offers both thermal processability and a high maximum use temperature (about 260 °C) [2].

Other fluoropolymers have been developed over the years, such as ETFE and polyvinylidene fluoride (PVDF). These polymers fall into the category of partially fluorinated polymers as their structure includes hydrogen atoms, as well as those of fluorine and carbon [3].

ETFE is an alternating copolymer (1:1 ratio) and an isomer of PVDF but it has a higher melting point [2].

The percentage of monomer present in the copolymer affects the structure and properties of the material, especially the degree of crystallinity and the melting point. As it is normally produced, ETFE has an alternation percentage of 88% with a T_m_ = 270 °C. A melting temperature of 300 °C is reached with 100% alternation [2].

These materials have high abrasion resistance over a wide range of temperatures and offer a good combination of tensile, impact and flexural strength, combining the properties of technopolymers with the chemical and thermal stability of perfluoro polymers [4,5,6,7]. The wear behavior can be improved by incorporating fillers, such as glass fibers. They are also self-cleaning materials.

ETFE is used in various fields, including that of architecture. The material can be extruded in very thin films (thickness of 50–300 µm), which can be used individually or to create multilayer coatings. These coatings, called cushions, are obtained by clamping several sheets at the edges and blowing air into the space between the films; these have found wide applicability in the construction sector, thanks to the advantages they are able to offer [3,8]. They are lightweight structures, allow the transmission of 90–97% of visible light, are good insulators, and have excellent flexibility. The most representative example is the Allianz Arena, a stadium in the city of Munich in Germany, the facades of which are covered with 66,500 m^2^ of cushions, for which the blowing pressure can be increased from 450 Pa to 800 Pa to allow the structure to withstand the weight of snow [9].

The combination of high strength and high transparency makes the ETFE films very suitable for protective purposes, thus ensuring continuous visits by the public, even during maintenance and/or restoration operations.

Nevertheless, to guarantee the efficiency of the film as a protective material, its properties must remain constant over time because restoration and maintenance works can be long-lasting, and its exposure to atmospheric events and pollution can provoke changes in the film’s chemical and physical characteristics [10].

In order to evaluate how ETFE films can be useful in this specific application, the present work focused on defining the mechanical resistance, especially the tearing and impact properties, before and after an accelerated aging process, in accordance with several studies about the resistance of blown cushions [11,12]. Indeed, by measuring the changes in the film after the aging treatment, chosen for representing a period of outdoor application close to the conditions of the real situation, the performance of the selected ETFE films can be assessed.

## 2. Materials and Methods

### 2.1. Samples

As samples, ETFE polymeric films with 95% transparency were chosen and the commercial polymers Moldflon (PFA, ErlingKlinger GmbH, Dettingen an der Erms, Germany) and Tefzel PV3221 (ETFE, DuPont, Meyrin, Switzerland) were identified, both having a thickness of 50 µm (Table 1). These polymers (both are melt-processed polymers) are characterized by their high processability [13].

The dimension of the films was selected in accordance with the standards of the mechanical tests employed before and after the accelerated aging process.

### 2.2. Artificial Ageing

Accelerated aging was carried out in a climatic test chamber (Angelantoni Industries S.r.l., Massa Martana, Italy). A mercury medium-pressure ultraviolet lamp (UV-A: 300–400 nm) with a maximum emission peak of 365 nm was used as a source of radiation. The total radiation that reached all the samples was 5.2 W/m^−2^, as measured by means of a radiometer (VLX-3W, Vilber, Collégien, France). The conditions inside the chamber were continuously monitored in terms of temperature and relative humidity [14,15,16], using a data logger (HOBO U10, Onset Computer Corporation, Bourne, MA, USA. The temperature range was from −20 to 70 °C, with an accuracy of ± 0.53 °C from 0 to 50 °C, and RH by 25% to 95%, with an accuracy of ±5% over the range of 5 to 55 °C). The setting of the aging cycles was defined to comply with ASTM D4329-21 [17] as the reference standard. The goal was to simulate the irradiation conditions in Rome for a period of two years. To this end, the SoDa Service was used as a reference. Indeed, the SoDa Service originates from a European project funded by the European Commission in 1999. The SoDa project was led by Mines ParisTech [18]; it comprises a database for estimating the average annual irradiance, used for agricultural applications and meteorological forecasts in different parts of the world.

Considering the exposure to irradiation over the Rome area, an average daily solar irradiance of 69,883.4 W/m^2^ was estimated to reach the Earth’s surface over five years (using data from 2000 to 2005). When sunlight passes through the atmosphere, all UV-C and most UV-B rays are absorbed by ozone, water vapor, oxygen, and carbon dioxide. UV-A is not filtered so significantly by the atmosphere (90–95% reaches the Earth’s surface), so its radiation accounts for 4193 W/m^2^ at the same time [19]. Furthermore, it shows the greatest interaction with organic materials and can induce degradation phenomena in the polymer structures [20,21].

Consequently, the conditions for the accelerated aging test were defined. The total irradiation for all the samples was 5.2 W/m^2^. The total exposure consisted of 6 cycles, performed as follows: 6-hour cycles of UV-A plus 3 h of condensation. In this way, it was possible to achieve the desired goal of simulating the radiation conditions over a period of two years in Rome.

The relative humidity value inside the chamber varied between 80 and 90%, while the temperature was maintained constantly at 25 °C. After each cycle, the samples were rotated horizontally in order to avoid the concentration of radiation in one area.

Furthermore, the films were exposed to UV-A radiation on white and black supports for evaluating the different conditions of irradiation and the interaction of light. Indeed, the experimental condition was optimized in the two borderline cases to identify whether the material under the film can absorb or emit radiation differently during exposure to UV-A, simulating its possible application on colored architectural and archaeological structures (e.g., decorative floors, mosaics, and frescoes).

For the sake of clarity, the following nomenclature (valid for both materials) is established:

- L, T: longitudinal and transverse specimens before the accelerated aging test.
- SB_L, SN_L: longitudinal specimens, aged using the white (SB) and black (SN) substrates, respectively.
- SB_T, SN_T: transverse specimens, aged using the white (SB) and black substrate (SN), respectively.

### 2.3. Materials Characterizations

This evaluation of the mechanical properties of polymeric films is a fundamental step in understanding the performance of a material. Indeed, in terms of the specific application considered in this study, the ETFE film needs to be considered resistant to tearing and tensile stresses and should not be damaged by falling objects. To this end, tensile, tear, and impact tests were performed to investigate the behavior of the films before and after aging. All specimens were conditioned and tested at 23 ± 2 °C and 50 ± 5% UR.

The films were also characterized by Fourier transform infrared (FT-IR) and differential scanning calorimetry (DSC) analysis before and after aging.

#### 2.3.1. Tensile Test

The tensile test was performed on a Zwick/Roell Z010 (Zwick/Roell GmbH, Ulm, Germany) universal testing machine, to comply with the standard, ASTM D 882 [22]. In detail, the setup of the instrument was as follows: a load cell of 1 kN; a preload of 3 N; test speed: 500 mm/min; initial grips distance: 100 mm; specimen size: 145 mm × 15 mm; and an initial gage length, L_0_, of 60 mm.

In accordance with the reference standard, ten longitudinal and ten transverse samples of each film were evaluated, reporting the average values and standard deviation of the following parameters: elastic modulus (MPa), tensile strength (MPa), yield strength (MPa), and percentage elongation at break (%).

#### 2.3.2. Tear Propagation Resistance

The test was performed on a Zwick/Roell Z010 (Zwick/Roell GmbH, Ulm, Germany) instrument, in compliance with the standard method for ASTM D 1938 (trouser tear test) [23]. In detail, the setup of the instrument was as follows: load cell: 1 kN; test speed: 250 mm/min; initial grips distance: 50 mm; specimen dimensions: 25 mm × 75 mm; cut length: 50 mm.

In compliance with the reference standard, ten longitudinal and ten transverse samples of each film were evaluated for measuring the trend of the force (N) as a function of the displacement (mm) and the critical energy T_c_ (N/m). More details of the test setup are reported in Supplementary Information (Section S1, Figure S1).

#### 2.3.3. Low-Velocity Impact Test

The instrumented impact test was performed on a CEAST/Instron 9340 (Instron, Pianezza, Italy), to comply with the standard ASTM D 7192. The film samples (8 cm × 8 cm) [24] were impacted with a hemispherical impactor of 16 mm diameter at different energy levels: 3.45 J, 1.54 J, and 0.38 J.

All tests were performed by placing the specimens on a ring-shaped support with a diameter of 40 mm.

#### 2.3.4. Chemical and Thermal Analysis

FT-IR analysis was performed directly on the films before and after aging using a Vertex 70 (Bruker Optics GmbH, Ettlingen, Germany) spectrometer, equipped with a diamond attenuated transmission reflection (ATR) cell. The ATR-FTIR spectrum was recorded with 512 scans in the mid-infrared range (400–4000 cm^−1^) at a resolution of 4 cm^−1^.

The DSC melting/cooling curves of non-aged and aged samples were performed using a TA DSC 2920 calorimeter (TA Instruments, New Castle, DE, USA) with a nitrogen-cooled cell and a flow rate of 70 mL/min. In all cases, the samples were first melted by heating up to 350 °C from 30 °C (5-min hold), then they were crystallized by cooling to 30 °C (5-min hold). In order to remove the thermal history of the samples, the thermal program was repeated twice with a heating rate of 10 °C/min. At least three analyses for each sample were carried out. Melting and crystallization temperatures were determined and the degree of crystallinity ($X_{c}$) of the samples was calculated, according to Equation (1):

(1) $$X_{c}\left(\%\right)=\frac{∆H_{m}}{∆H_{m}^{0}}\cdot 100$$

where $∆H_{m}$ represents the experimental enthalpy of the melting of the sample (J/g), and $∆H_{m}^{0}$ represents the enthalpy of melting for 100% crystalline ETFE or PFA (J/g), taken as 113.4 [25,26] and 38.8 J/g [27], respectively.

## 3. Results and Discussion

### 3.1. Tensile Test

**Figure 1:**
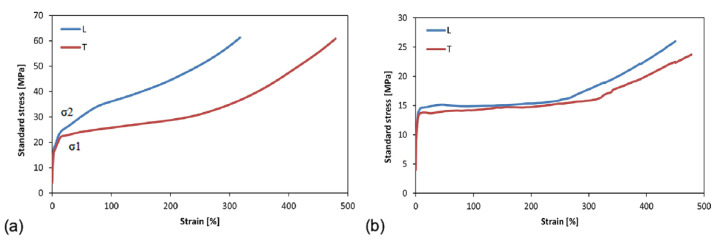
Typical stress-strain curves for the ETFE (**a**) and PFA (**b**) non-aged film. L = longitudinal specimen, T = transverse specimen.

Figure 1 shows the typical stress-strain curves of the ETFE and PFA films.

The films were analyzed in both the longitudinal and transverse directions, reporting a different trend in the two cases due to their anisotropic behavior.

ETFE demonstrated the behavior of a semicrystalline polymer (Figure 1a): a linear elastic trend, followed by yielding and strengthening upon large deformations. In particular, higher percentage elongation at break was recorded for transverse specimens (greater than 400%). The production process, i.e., cast films, is responsible for the observed anisotropy as the macromolecules are aligned in the process direction.

Furthermore, the phenomenon of double-yielding (as previously described in the literature [28,29]) is observed. Indeed, the double yield can be traced back to a reorganization of the crystal structure (σ1), followed by its fragmentation (σ2). Therefore, the presence of a double yield indicates a change in the stiffness of the material. The σ1 is considered the point where the linear deformation ends, and this first section is used to determine the elastic modulus.

The values of σ1 and σ2 for the non-aged L and T specimens are within the values reported by the DuPont technical data sheets and remain around 15 MPa and 20 MPa, respectively (Table 2).

The PFA films also exhibited behaviors typical of semi-crystalline polymers (Figure 1b), with extensive ductility and toughness in both the longitudinal and transverse directions, confirming a slight anisotropic response. In Table 3, the values of percentage elongation at break, yield strength, and tensile strength for non-aged PFA films are reported.

**Figure 2:**
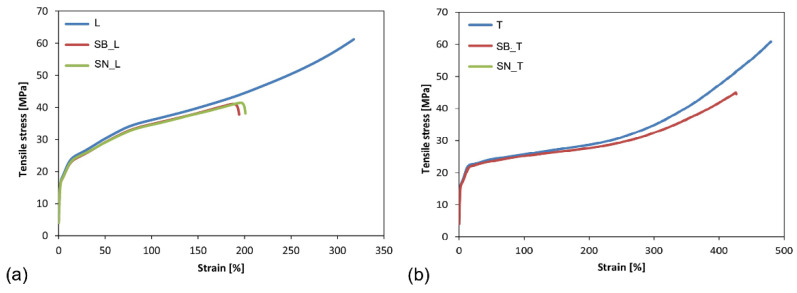
Comparison of the stress-strain curves before and after aging for L (**a**) and T (**b**) ETFE specimens on the white (SB) and black (SN) substrates. The SN_T curve is superimposed on the SB_T curve.

After the aging of the ETFE film, the shape of the stress-strain curve is not significantly affected, with the exception of a reduction in the overall ductility and tensile strength, as shown in Figure 2.

From the results, it appears clear that the color of the support did not affect the resulting tensile response, while the phenomenon of double yielding was maintained and the values of σ1 and σ2 remained almost unchanged (Table 2). The strength decreased after the aging cycle, remaining the same for the longitudinal and transverse specimens. In particular, the measured values dropped from 60 MPa to 42 MPa, a decrease of about 30%.

The percentage deformation at break experienced an evident decrease (16%); however, it maintained a higher value in the case of the transverse specimens (both before and after the aging treatment). For the longitudinal specimens, on the other hand, the decrease in ductility was more evident (34%), especially for those specimens aged on the black substrate (Table 2).

If the tensile strength and ductility decreased in both categories of films analyzed (L and T), the same is not true of the elastic modulus, which was not significantly influenced by the aging treatment, irrespective of the substrate used. Indeed, an increase in E for the L specimens and a decrease for the T specimens was observed. In both cases, however, there were no differences between the films on the black or the white substrates. To be specific, the elastic modulus increased by about 8% for the L specimens and decreased by 5% for the T specimens.

After aging, the behavior exhibited by the PFA films was characterized by a high dispersion of data; some specimens showed low elongation at break (30%), and others, an elongation at break up to 350% (these performances were obtained for both longitudinal and transverse films). This trend was also confirmed with a second series of samples. Despite this, thanks to the tests carried out with the strain gauge, the elastic modulus both before and after the aging cycle was evaluated; the mean values and standard deviations are included in Table 4.

The elastic modulus values obtained for the PFA films are lower than those of the ETFE films. Furthermore, there are no significant differences between the E values obtained after aging using either the black or the white substrates. In both cases, the decrease in the elastic modulus is about 4%.

### 3.2. Trouser Tear Test

**Figure 3:**
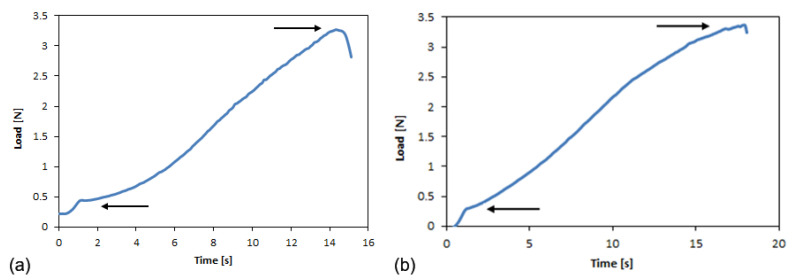
Load-time curves for L (**a**) and T (**b**) ETFE specimens: the points of the initial tear load and maximum load are indicated.

The typical load-time curves for the longitudinal and transverse ETFE specimens reported in Figure 3 highlight the response of a highly extensible material, as no constant load during trouser tear testing was observed, while the deformation energy of the specimen legs was significantly higher than the tearing energy. In particular, two points are highlighted: the initial tear load and the maximum load. This behavior is retained even after aging.

**Figure 4:**
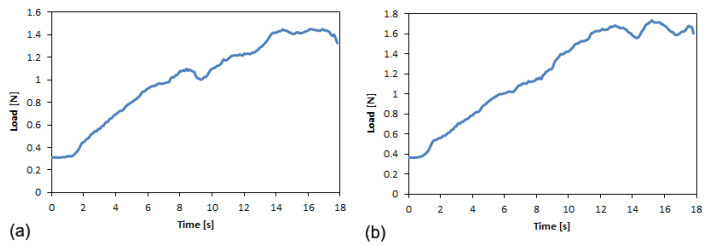
Typical load-time curves for PFA in longitudinal (**a**) and transverse (**b**) specimens.

For the PFA films, the load-time curves are reported in Figure 4. The trends that are reported are different from those obtained for the ETFE films: the maximum load point is not well defined, nor is the initial tear load point. After a short interval of time, these samples exhibited a constant load during trouser tear testing. In this case, an average tear force is defined.

**Figure 5:**
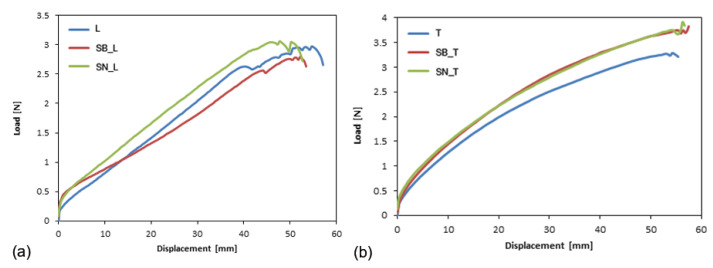
Typical load-displacement curves for longitudinal (**a**) and transverse (**b**) ETFE specimens, before and after aging.

In Figure 5, the trends of the load-displacement curves for the longitudinal and transverse ETFE specimens as a function of aging on black and white substrates are shown.

The L and T specimens featured different damage modes. In the T specimens, the propagation of the crack followed the trajectory of the pre-crack in a straight line.

**Figure 6:**
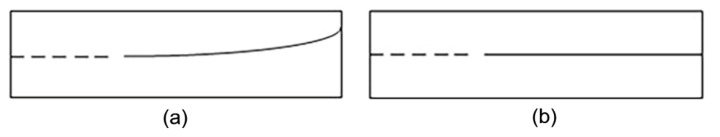
Schematic diagram of the fracture line exhibited by longitudinal (**a**) and transverse (**b**) specimens.

In the case of longitudinal specimens, as soon as the test began, the specimen was rotated by 90 °C, and failure testing continued under a tensile load. Indeed, the specimens examined after the test showed a curvilinear fracture line (J-Type) (Figure 6), indicating that the material did not break due to tearing (see Supplementary Information, Section S2, Figures S2–S4).

After aging, the behavior of the material did not change, and the same J-type failure was observed for the longitudinal specimens (Figure 5a). Therefore, for these samples, it was not possible to evaluate the critical fracture energy, as indicated in the literature for other ultra-thin polymer films characterized by the same behavior [30].

Furthermore, no differences were noticed in response to the white and black substrates.

In Figure 5b, the complete correspondence between the SB_T and SN_T curves can be observed, showing a value slightly greater than for the non-aged samples, due to the aging treatment.

**Figure 7:**
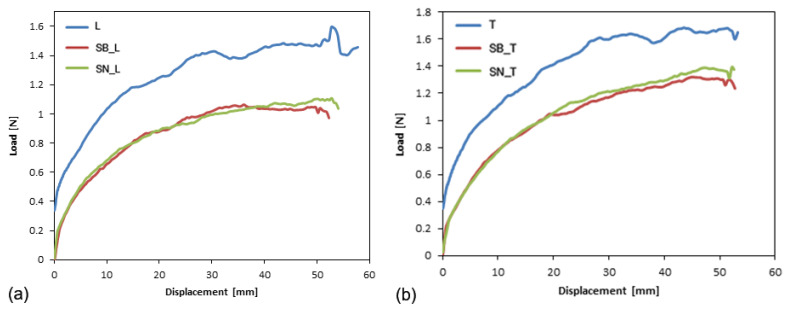
Typical load-displacement curves before and after aging for PFA longitudinal (**a**) and transverse (**b**) films.

In the case of PFA film, the constant load behavior observed during the trouser test is also shown in the load-displacement curves for the L and T films (Figure 7).

Indeed, two distinct regions can be identified for both curves:

- Alignment of the legs of the specimen, characterized by an increase in the tear force exerted, and formation of the fracture surface around the tip of the notch.
- Stabilization of the tear force around an average value, until the film breaks (the curve almost plateaus).

The average force value is reached when the extension exceeds 20 mm for both cutting directions of the specimens. This average value coincides with the force, F, used for calculating the critical fracture energy.

The fracture lines in the two cases (L and T) are similar, without any curvilinear trend as observed in longitudinal ETFE films (Figure 5a). Indeed, the crack propagation continued along the direction of the notch, maintaining a straight path.

After the accelerated aging cycle, the load-displacement curves related to the tear tests are shown in Figure 7.

Observing the curves, a complete correspondence exists between the aged films using the black and white substrates, while a significant decrease in average strength is observed after the aging cycle.

In Supplementary Information (Section S1), the equations used for the calculation of the critical fracture energy (Tc) are reported. Taking into account the high extensibility found for the ETFE films, the deformation energy density was taken into account for the calculation of Tc. Indeed, when a material is classified as highly extensible, the deformation energy is greater than that of a tearing one; therefore, other deformation modes are involved [23]. Considering the load-displacement curves, the obtained maximum displacement was 50 mm (i.e., two times the length of the non-pre-stressed area), and the value used for the calculation of Tc λ was 1.

The values obtained are shown in Table 5, where only the Tc values of the T-samples are reported for the ETFE films due to the J-type behavior of the L-samples, as discussed above.

For calculating the critical fracture energy for PFA polymer, the extension reached is 50 mm and is, therefore, equal to the maximum level that is allowable. Consequently, the behavior of the film is considered similar to that of non-extensible materials, even if the plateau is not well defined (as reported elsewhere for other films, e.g., polycarbonate [31]). The average values and standard deviations of the tearing force and critical tear energy for both films are shown in Table 5.

### 3.3. Impact Test

**Figure 8:**
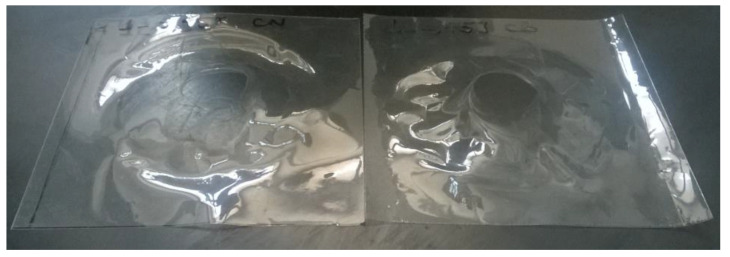
Specimens of ETFE after the impact test at Energy = 3.45 J.

**Figure 9:**
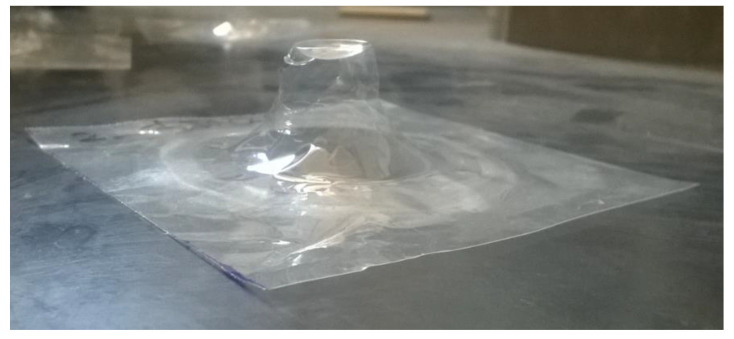
Specimen of ETFE after the impact test, at Energy = 1.54 J.

**Figure 10:**
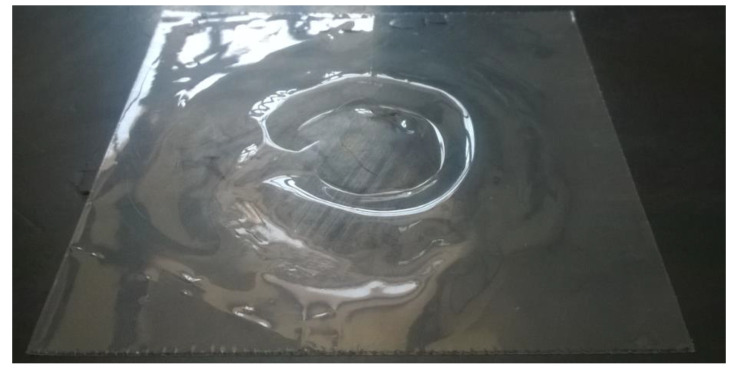
Specimen ETFE after the impact test at Energy = 0.38 J.

ETFE film samples were analyzed considering three different impact energies: 3.45 J, 1.54 J, and 0.38 J (Figure 8, Figure 9 and Figure 10).

The resistance offered by the specimens is investigated by studying the following trends: Load (N)—Deflection (mm), and Energy (J)—Time (ms), which are given in the Supplementary Information, Section S3, Figures S5–S12.

Table 6 shows the average values and standard deviations of the maximum force, the corresponding displacement, and the total energy absorbed before and after aging on different substrates.

From the reported values, the peak force and absorbed energy were found to decrease after aging, and, in this case, the type of substrate did not play a significant role. At 3.45 J and 1.54 J, all the specimens were perforated (Figure 8 and Figure 9), while it was only at 0.38 J that a rebounding of the impactor was detected (Figure 10), along with significant plastic deformation. The differences in impact resistance between virgin and aged samples were limited, thus suggesting a non-dramatic degradation of their response under impact loading, as was already detected in the quasi-static case.

In the case of the PFA films, all the samples were perforated at each impact energy level. Considering the fact that no differences between behavior were observed with the white or black substrate, only the aged specimens on the white substrate were analyzed. All curves are included in the Supplementary Information, Section S3.

**Figure 11:**
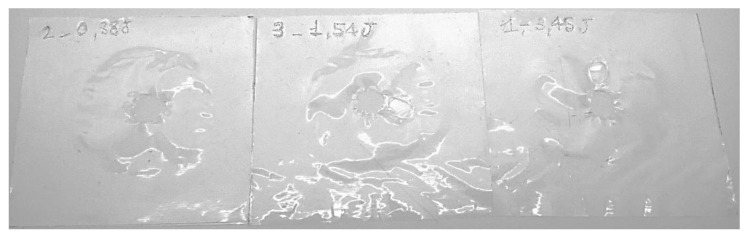
PFA films after the impact test: the film was perforated at all three tested impact energies.

Table 7 summarizes the average values and standard deviations of the main impact test results, while Figure 11 shows the specimens after the test (the samples demonstrated the same failure mode for the three impact energies).

### 3.4. FTIR and DSC Analysis

**Figure 12:**
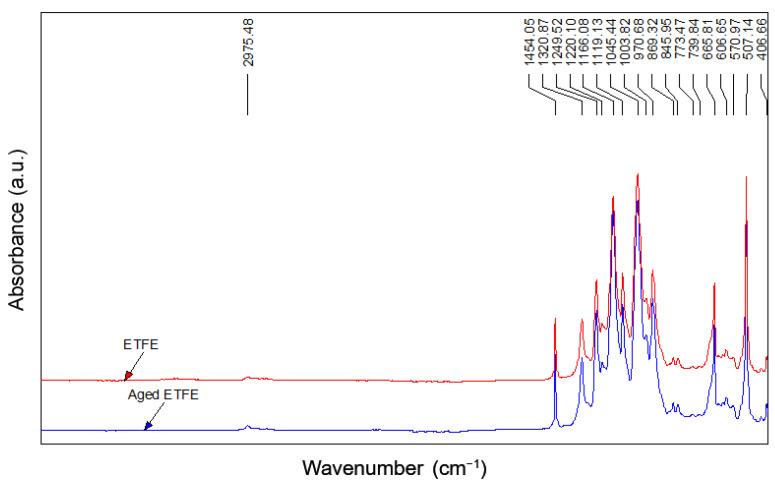
FTIR spectra of ETFE films before and after aging.

**Figure 13:**
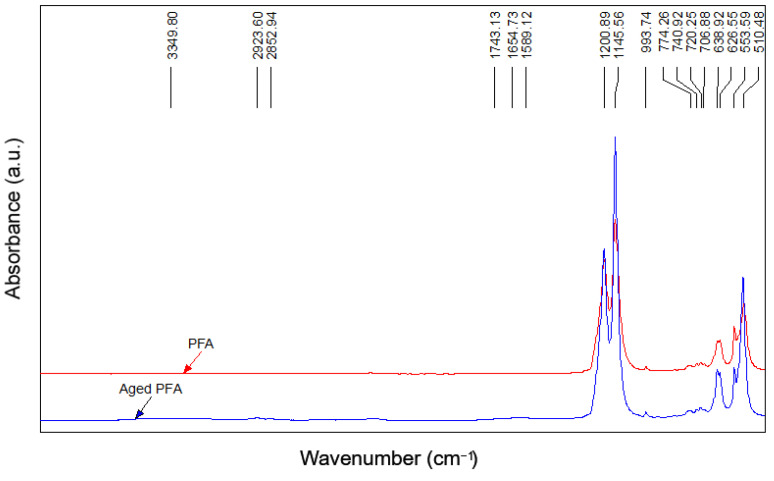
FTIR spectra of PFA films before and after aging.

In Figure 12 and Figure 13, the ETFE and PFA spectra before and after aging are shown.

In the ETFE spectra, the typical vibrations of the CF_2_ are present in the range of 1300–1000 cm^−1^. The stretching vibration at 2975 cm^−1^ and the bending vibration at 1454 cm^−1^ are related to the presence of CH_2_ groups, confirming the typical–[CH_2_–CH_2_–CF_2_–CF_2_]_n_– polymeric structure [32]. Before and after aging, no changes are evident, and the spectra are perfectly overlapped.

**Figure 14:**
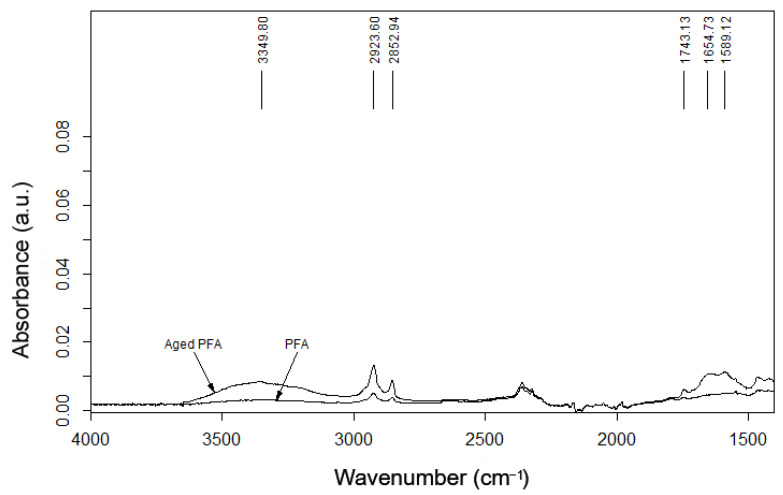
The 4000–1400 cm^−1^ range of the spectrum relating to unaged and aged PFAs.

In the PFA film, the main effects shown are due to the CF_2_ vibrations (at 1200 and 1145 cm^−1^), but, after aging, the spectrum (given in Figure 14) showed the presence of OH groups (3349 cm^−1^) and the absorbance of C=O groups (1743 cm^−1^, 1654 cm^−1^, and 1589 cm^−1^) related to the COOH groups, due to the degradation of the molecular chains during the aging process [12].

**Figure 15:**
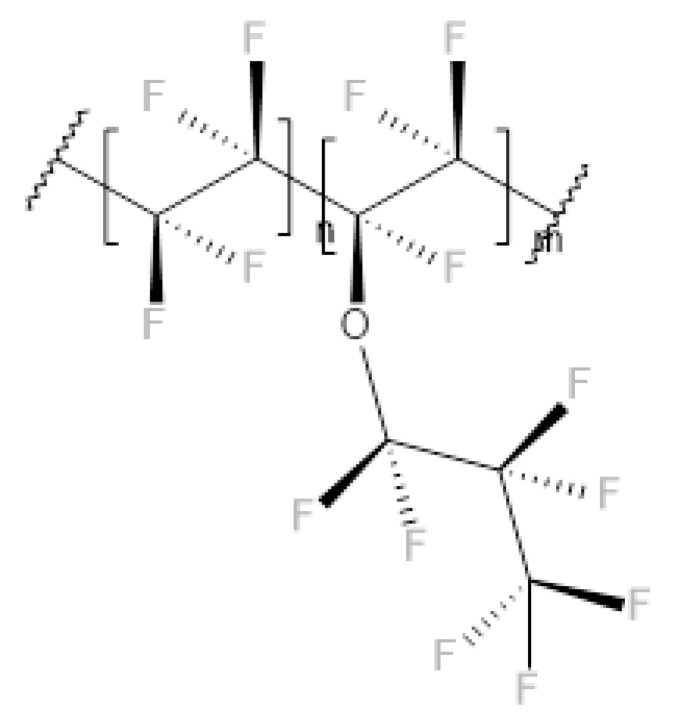
Chemical structure of the PFA.

The behavior of the aged PFA can be explained by considering the chemical structure of the polymer, which is schematically represented in Figure 15. Indeed, the ether group can be attacked during the aging process and COOH groups can be introduced [12].

The FTIR analysis suggests a lower resistance to aging in PFA films compared to ETFE, showing the initial degradative oxidation state of its polymeric structure, which can affect its mechanical behavior over time, as confirmed by the tensile and tear tests.

DSC was employed to study the effect of the absorbed UV radiation on the thermal properties of the polymer matrices. In Table 8, the comparison of the first melting cycle (Tm1) of non-aged and aged films shows the effect of irradiation on the structural changes and on the thermal history of the considered samples. The cooling (Tc) and second heating (Tm2) cycle provide information about the effect of irradiation on the crystallization of the polymer chain and on the final crystallinity ($X_{c}$). Compared to unaltered polymer, it can be assumed that both aged samples primarily underwent chain scission when irradiated with UV light. Indeed, a decrease in molecular weight resulted in better packing of the polymer chains, which leads to the formation of more thermally stable crystals that melt at a higher temperature [32]. This behavior was confirmed by a decrease in crystallinity ($X_{c}$), probably due to the interruption of the more linear sequences of the polymeric matrix resulting from a process of chain scission and/or crosslinking of the generated fragments.

## 4. Conclusions

The covering and protection materials and methods used in the area of conservation of archaeological assets are constantly evolving. In this work, the properties of fluoropolymer films such as ETFE have been studied, to understand whether these materials can be used in this context. Establishing their performance after a certain period of environmental exposure required specific mechanical analyses before and after undergoing an aging treatment. Furthermore, specific analysis, such as the trouser tear test and impact test, can help the assessment of the exposure performance of these films in strong winds or storms. Knowledge of these properties is necessary for archaeological sites where precious artifacts (such as mosaics or painted walls) are present and need to be protected. Indeed, exposure to atmospheric agents (e.g., rain and wind) can destroy monumental heritage sites, and their protection can be achieved by covering them up. However, covering such sites blocks this cultural heritage from view, creating difficulties for the visitors and economic problems for the management and funding of the site. 

Considering the mechanical tests carried out, ETFE films do not show great differences after aging; in fact, their elastic modulus is reduced by only ≈ 5% and is considerably higher than PFA films (1075.38 MPa for ETFE and 625.48 MPa for PFA). Regarding the results obtained from the tearing tests, the two films demonstrated a strong difference in their behavior. Indeed, in terms of the ETFE film, the load increased continuously; conversely, with the PFA film, the load value reached a particular level, remaining almost constant until the specimen was broken. A slight reduction of about 10% in the critical tear energy values for PFA films was observed after aging. Furthermore, in the impact test, no change before and after aging was observed in the two polymer films; however, the ETFE film showed better peak force than the PFA film (82.95 N and 42.22 N, respectively).

To summarize, both before and after accelerated aging, the ETFE Tefzel PV3221 film showed the best performance and represents the best candidate for further analyses in this specific field of application.

In addition, the use of these films in the conservation areas of archaeological sites can be of help where the use of protective paints might cause damage and cannot be applied. A prolonged study of these films can therefore extend their possible field of application in the conservation of cultural heritage. 

## Figures and Tables

**Table 1:** Properties of commercial ETFE films.

Sample	MFR ^1^ (g/10 min)	Melting Temperature ^2^ (°C)	Water absorption ^3^ (24 h)
PFA	4	315	<0.01
ETFE	7	327	<0.01

^1^ ASTM D3159; ^2^ ASTM D3418; ^3^ ASTM D570.

**Table 2:** E, σ, σ1, σ2, and ε for the non-aged and aged L and T specimens of ETFE polymer.

Sample	E (MPa)	σ (MPa)	ε (%)	σ1 (MPa)	σ2 (MPa)
L	1075.38 ± 43.51	60.38 ± 7.91	310.74 ± 45.55	15.42 ± 0.78	22.60 ± 0.90
T	1033.50 ± 42.71	59.64 ± 3.56	478.81 ± 17.01	14.86 ± 0.55	20.51 ± 0.55
SB_L	1111.30 ± 62.06	42.96 ± 5.72	207.23 ± 45.98	15.70 ± 0.70	21.44 ± 0.58
SB_T	980.00 ± 40.91	42.28 ± 3.76	402.55 ± 38.28	14.36 ± 0.50	20.16 ± 0.25
SN_L	1101.46 ± 78.5	42.41 ± 2.35	197.99 ± 25.28	15.32 ± 0.21	20.91 ± 0.66
SN_T	981.23 ± 39.89	42.38± 3.05	401.99 ± 37.28	14.55 ± 0.30	20.43 ± 0.40

**Table 3:** Mean values and standard deviation for PFA specimens after tensile tests.

Sample	E (MPa)	σ (MPa)	ε (%)	σ_y_ (MPa)
L	625.48 ± 45.91	23.38 ± 2.62	405.41 ± 49.01	12.20 ± 1.31
T	643.23 ± 30.85	20.74 ± 2.70	418.63 ± 56.78	11.80 ± 1.07

**Table 4:** Young’s modulus values for longitudinal (L) and transverse (T) PFA films after aging on white (SB) and black (SN) supports.

Sample	E (MPa)
SB_L	592.64 ± 12.03
SB_T	610.06 ± 24.41
SN_L	605.79 ± 28.30
SN_T	607.92 ± 11.60

**Table 5:** Properties of the ETFE and PFA trouser tear test; mean values and standard deviations are reported.

Sample	Tearing Force (N)	Tc (N/mm)
	**ETFE**	
L	3.03 ± 0.18	-
T	3.30 ± 0.08	131.70 ± 3.30
SB_L	2.89 ± 0.21	-
SB_T	3.76 ± 0.24	150.67 ± 9.62
SN_L	3.10 ± 0.17	-
SN_T	3.77 ± 0.17	151.04 ± 6.60
	**PFA**	
L	1.32 ± 0.07	52.90 ± 2.14
T	1.47 ± 0.03	59.06 ± 1.20
SB_L	0.96 ± 0.00	38.29 ± 1.54
SB_T	1.14 ± 0.05	45.55 ± 1.98
SN_L	0.97 ± 0.05	38.74 ± 2.14
SN_T	1.19 ± 0.06	47.61 ± 2.53

**Table 6:** Average values and standard deviations related to the results obtained in the impact test for ETFE, before and after aging.

Sample	Impact Energy (J)	Impact Velocity (m/s)	Peak Force (N)	Peak Displacement (mm)	Total Energy (J)
Non-aged	3.45	2.50	117.70 ± 3.34	19.66 ± 0.74	2.26 ± 0.05
SB			109.04 ± 2.36	18.63 ± 2.35	1.38 ± 0.33
SN			109.70 ± 7.92	19.16 ± 3.05	1.49 ± 0.3
Non-aged	1.54	1.67	113.71 ± 4.45	18.82 ± 1.7	1.73 ± 0.04
SB			106.12 ± 2.66	20.89 ± 1.15	1.64 ± 0.02
SN			108.60 ± 0.67	21.60 ± 0.23	1.68 ± 0.08
Non-aged	0.38	0.83	82.95 ± 1.15	10.70 ± 0.22	0.50 ± 0.01
SB			74.40 ± 1.35	11.02 ± 0.04	0.46 ± 0.01
SN			77.02 ± 1.36	10.28 ± 0.18	0.45 ± 0.01

**Table 7:** Average values and standard deviations for the three impact energies, for PFA non-aged and aged films.

Sample	Impact Energy (J)	Impact Velocity (m/s)	Peak Force (N)	Peak Displacement (mm)	Total Energy (J)
Non-aged	3.45	2.50	40.36 ± 3.40	9.78 ± 0.54	0.30 ± 0.06
SB			42.52 ± 0.73	12.87 ± 3.80	0.34 ± 0.06
Non-aged	1.54	1.67	42.87 ± 1.83	9.64 ± 0.34	0.36 ± 0.05
SB			41.52 ± 2.41	10.36 ± 0.36	0.33 ± 0.12
Non-aged	0.38	0.83	42.22 ± 0.34	12.58 ± 3.93	0.35 ± 0.01
SB			40.11 ± 4.52	9.07 ± 0.22	0.33 ± 0.04

**Table 8:** Melting temperature, melting enthalpy, and the crystallinity of non-aged and aged films.

Sample	Tm1 (°C)	∆H_m_ (J/g]	Tm2 [°C]	∆H_m_ [J/g]	Tc [°C]	$X_{c}$ [%]
ETFE	259.69	29.47	259.97	42.89	245.45	37.82
ETFE Aged	262.68	28.59	260.70	39.58	245.55	34.90
PFA	313.97	24.18	313.93	30.95	291.93	79.77
PFA Aged	314.71	21.99	314.80	27.91	291.22	71.93

## Data Availability

The raw/processed data required to reproduce these findings are available on request.

## Supplementary Materials

The following supporting information can be downloaded at: https://www.mdpi.com/article/10.3390/polym14050912/s1, Figure S1: Details from the trouser tear test of an aged PFA T specimen; Figure S2: J-type cracks for unaged Longitudinal ETFE samples; Figure S3: Cracks for the unaged Transverse ETFE samples; Figure S4: Cracks for Longitudinal and Transverse unaged PFA samples; Figure S5: Load-Deflection curves for unaged ETFE sample for three impact energies; Figure S6: Energy-Time curves for unaged ETFE for the three impact energies; Figure S7: Load-Deflection curves for aged ETFE sample for the three impact energies; Figure S8: Energy-Time curves for aged ETFE sample for the three impact energies; Figure S9: Load-Deflection Curves for unaged PFA sample for three impact energies; Figure S10: Energy-Time curves for unaged PFA sample for three impact energies; Figure S11: Load-Deflection Curves for aged PFA samples for three impact energies; Figure S12: Energy-Time curves for aged PFA samples for three impact energies.
