# Supplementary material for: Effect of Aging on the Mechanical Properties of Highly Transparent Fluoropolymers for the Conservation of Archaeological Sites
Source: Polymers (Basel). 2022 Feb 24;14(5):912. doi: 10.3390/polym14050912 (PMC8912537; doi:10.3390/polym14050912)
Supplement: Supplementary file 1 [file polymers-14-00912-s001.zip › polymers-1559617-supplementary.pdf]

polymers

MDPI

Supplementary Material

Effect of Aging on the Mechanical Properties of Highly Transparent Fluoropolymers for the Conservation of Archaeological Sites

Maria Paola Bracciale *, Lorena Capasso, Fabrizio Sarasini, Jacopo Tirillò and Maria Laura Santarelli *

Department of Chemical Engineering Materials Environment (DICMA), University of Rome Sapienza, Via Eudossiana 18, 00184 Rome, Italy; lorena.capasso@gsk.com (L.C.); fabrizio.sarasini@uniroma1.it (F.S.); jacopo.tirillo@uniroma1.it (J.T.)
* Correspondence: mariapaola.bracciale@uniroma1.it (M.P.B.); marialaura.santarelli@uniroma1.it (M.L.S.)

Citation: Bracciale, M.P.; Capasso, L.; Sarasini, F.; Tirillò, J.; Santarelli, M.L. Effect of Aging on the Mechanical Properties of Highly Trans-parent Fluoropolymers for the Conservation of Archaeological Sites. *Polymers* **2022**, *14*, 912. https://doi.org/10.3390/polym14050912

Academic Editor: Paulo Nobre Balbis dos Reis

Received: 27 January 2022
Accepted: 11 February 2022
Published: 24 February 2022

Publisher's Note: MDPI stays neutral with regard to jurisdictional claims in published maps and institutional affiliations.

Copyright: © 2022 by the authors. Licensee MDPI, Basel, Switzerland. This article is an open access article distributed under the terms and conditions of the Creative Commons Attribution (CC BY) license (http://creativecommons.org/licenses/by/4.0/).

S1. Trouser Tear Test method

The method consists in making a notch in the polymer film, which acquires the characteristic trouser shape giving the name to the method (Figure S1).

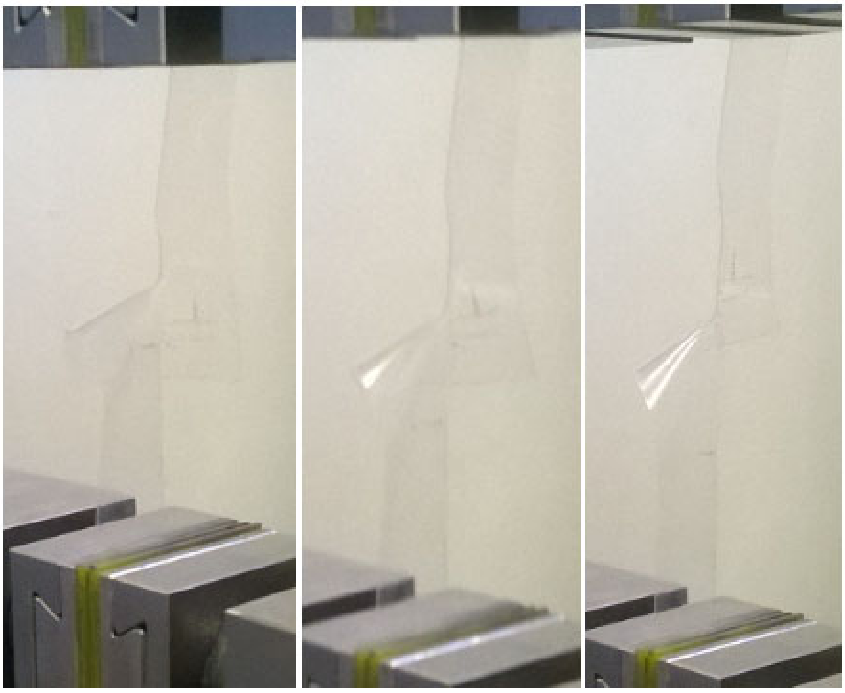

Figure S1. Details from the trouser tear test of an aged PFA T specimen.

The description of fracture includes three stress modes that determine crack propagation [1]:

- Mode I: opening.
- Mode II: in-plane shear.
- Mode III: out-of-plane shear

The third mode of fracture is one of the most common ones in polymeric films and thanks to this test the tear resistance offered by these films can be evaluated.

The performance offered by the films during the test depends on several factors and in order to better characterize the materials, the trajectory of the crack propagation has to be taken into account, but also the thickness of the film and the speed of load application

during the test. The effects of the orientation of the film, the geometry and the fracture mechanism must therefore be discussed [2].

Especially, by observing the Load - Time curves, low or high extensible films can be distinguished.

In the case of ductile polymeric films, two of the major difficulties are the possible deformation of the "legs" of the specimen and of the area around the crack, thus recording the presence of other fracture modes. To understand if other phenomena are involved, the trend of the force applied as a function of the elongation of the specimen (evaluated as the distance between the crosspieces) must be observed.

The breakout energy is defined as the energy released per unit area of the surface of the propagating crack:

$$T = - (\partial W / \partial A) \tag{S1}$$

where T is the energy released, W the elastic energy stored by the specimen and A the fracture area [3].

The tear energy is considered the driving force for the propagation of the crack and includes the surface energy, the energy dissipated in the plastic flow and that dissipated in the viscoelastic process. When it reaches the critical value, Tc, crack growth is observed. The equations used for the calculation of Tc have been derived from experiments on rubber, but they are also applicable for polymeric films [1]. Furthermore, the trouser tear test has an advantage: the tear energy depends only on the length of the crack and on the deformation in the area around the crack itself, while it is independent of the way in which the force is applied.

The calculation differs in the case of low and high extensible films. In the first case, the following relationship can be used for the calculation of the critical fracture energy:

$$Tc = 2F / B \tag{S2}$$

where F is the force and B the thickness of the specimen.

In the case of extensible specimens, on the other hand, Tc is evaluated as follows:

$$Tc = (2F\lambda) / B - wE \tag{S3}$$

where λ is the ratio between the final length of the specimen leg and the initial length, w the initial leg width and E the strain energy density [N / mm^2].

For this tear behavior, it is important to observe how much the deformation of the legs is relevant: if the weight of the deformation is minimal, (S2) can be used for the calculation of Tc. In this way, the critical fracture energy depends solely on the thickness, and not on the size of the sample.

Furthermore, the work spent during the test corresponds to the area below the Load - Displacement curve.

S2. Behavior of the samples during the Trouser Tear Test

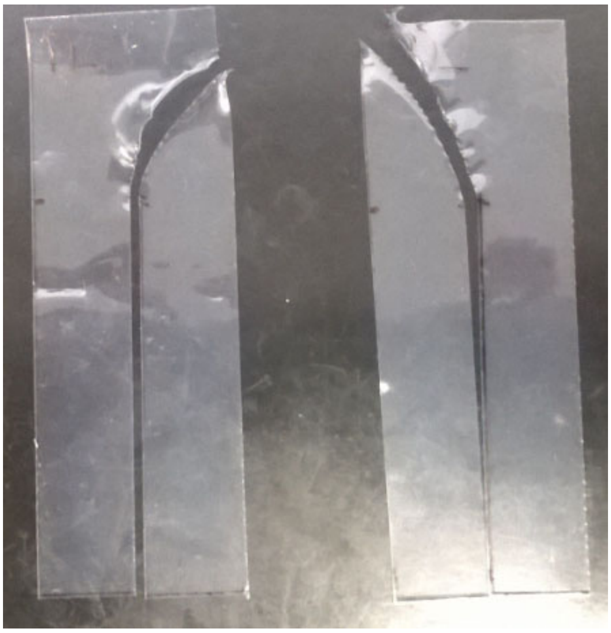

Figure S2. J-type cracks for unaged Longitudinal ETFE samples.

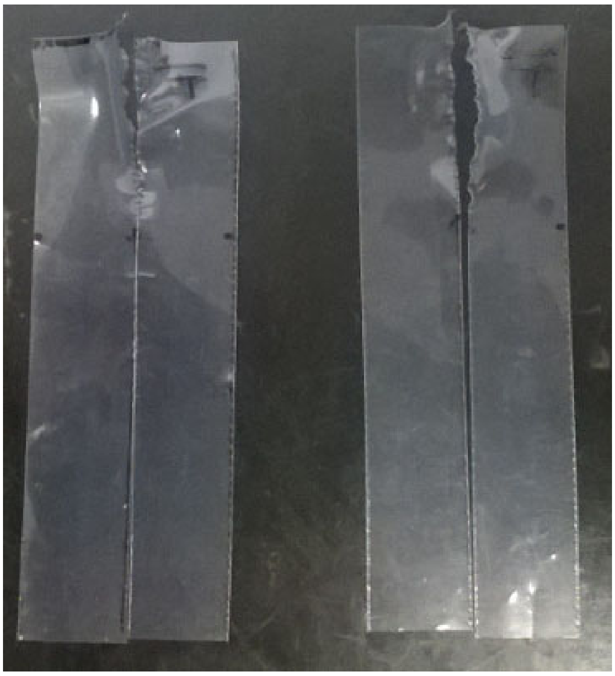

Figure S3. Cracks for the unaged Transverse ETFE samples.

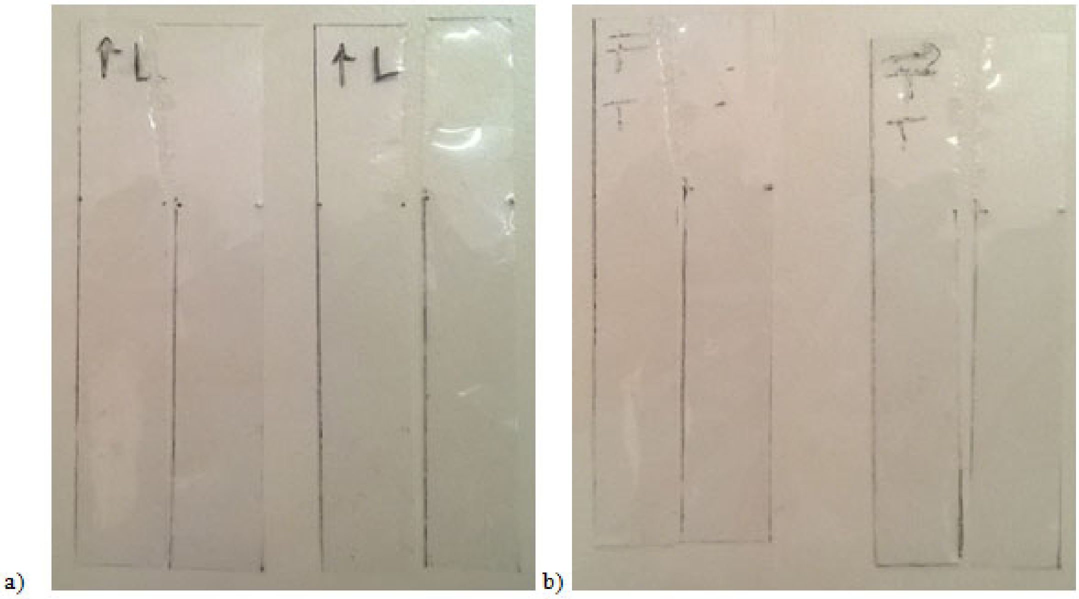

Figure S4. Cracks for Longitudinal and Transverse unaged PFA samples.

S3. Impact test results

Figure S5 shows the Load - Deflection trends for the three different impact energies (0.38J, 1,54J, 3,45J) in unaged ETFE samples.

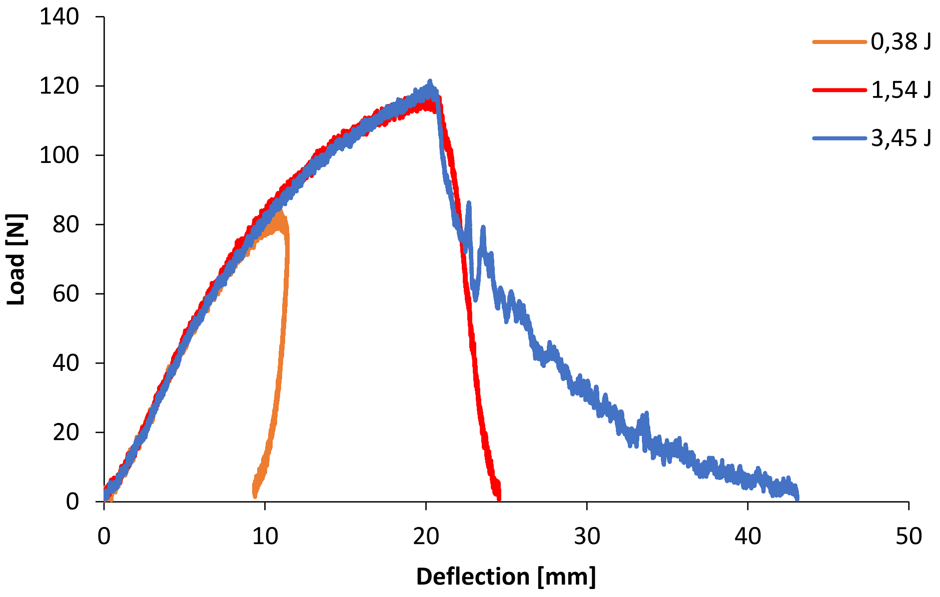

Figure S5. Load – Deflection curves for unaged ETFE sample for three impact energies.

Observing the figure, the higher impact energies, 3.45 J and 1.54 J are used to perforate the films. Indeed, a complete rupture occurs in the case of the impact energy of 3.45 J and lateral rupture for 1.54 J.

In the third case, however, the specimen does not break, and a rebound of the dart was observed.

Figure S6 shows the Energy - Time trend for the three impact energies. For higher energies, after an increase in energy, a stationary value (a plateau) is observed, maintained

until the films break. In the case of the lowest impact energy, however, part of the energy is released. Furthermore, the film breaks before it can absorb all the energy related to the impact; in the other two cases the material absorbs the entire impact energy.

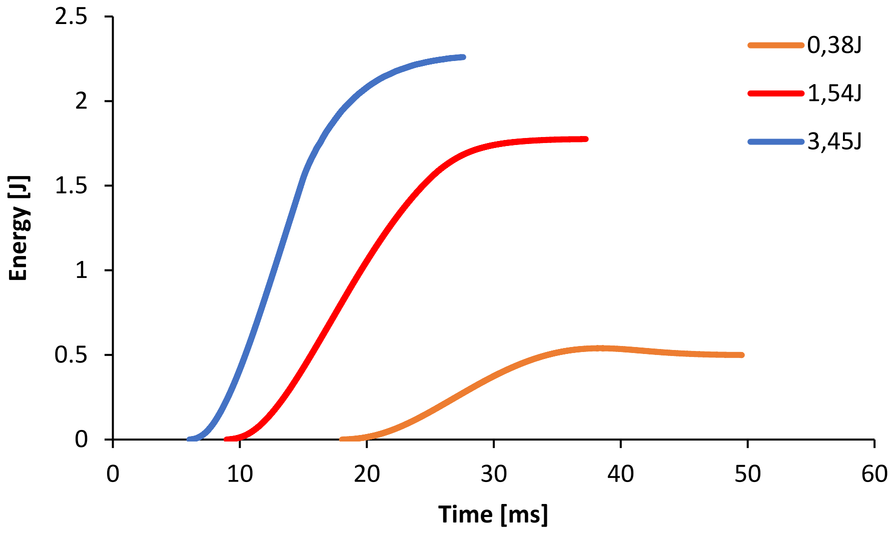

Figure S6. Energy - Time curves for unaged ETFE for the three impact energies.

After the accelerated aging cycle by UV-A irradiation, the Load - Deflection and Energy - Time curves are shown in Figures S7 and S8 for the three impact energies.

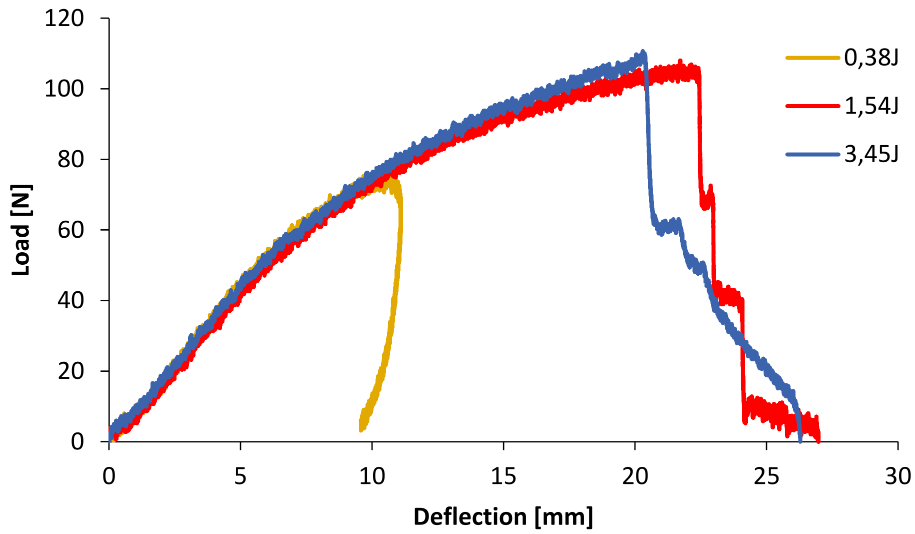

Figure S7. Load – Deflection curves for aged ETFE sample for the three impact energies.

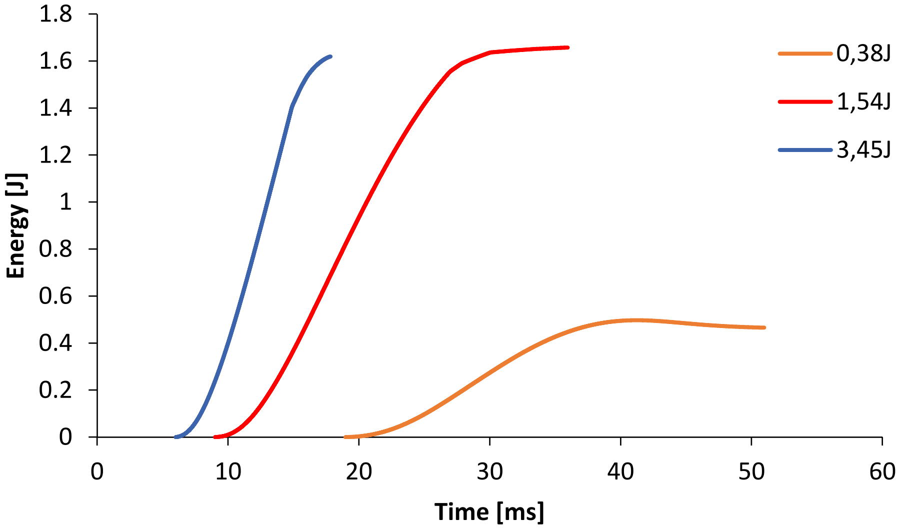

Figure S8. Energy - Time curves for aged ETFE sample for the three impact energies.

In Figure S9, the Load - Deformation curves for the three impact energies are shown. Considering the Energy - Time curves (Figure S10), the total energy absorbed is not equal to the impact energy, but lower (about 0.3 J). Indeed, the film breaks before that the relative impact energy is absorbed.

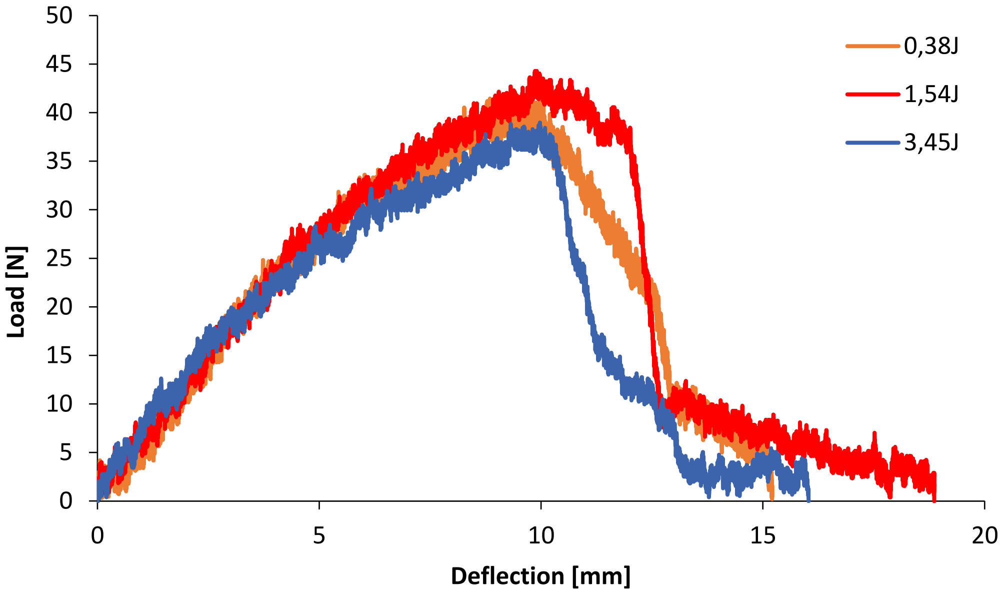

Figure S9. Load – Deflection Curves for unaged PFA sample for three impact energies.

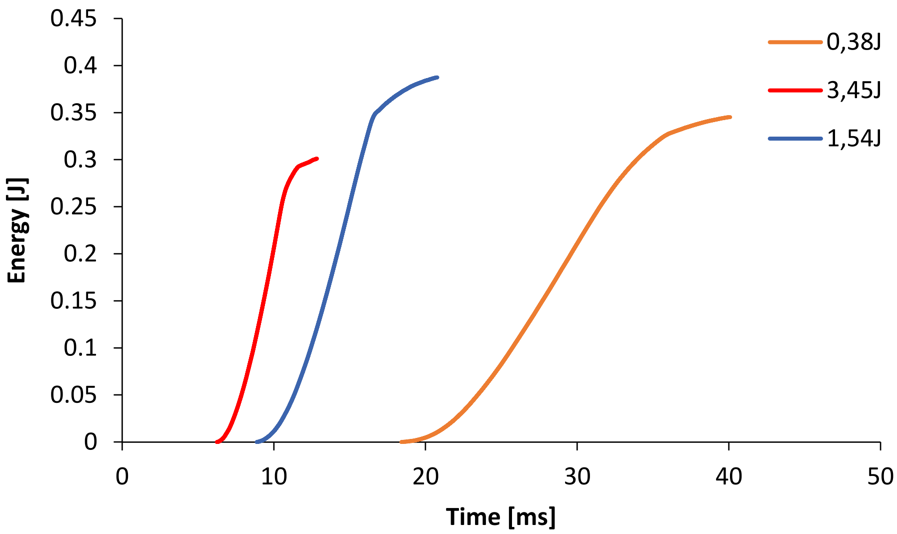

Figure S10. Energy - Time curves for unaged PFA sample for three impact energies.

After aging, the impact curves (S11 and S12) showed a similar behavior to the unaged samples.

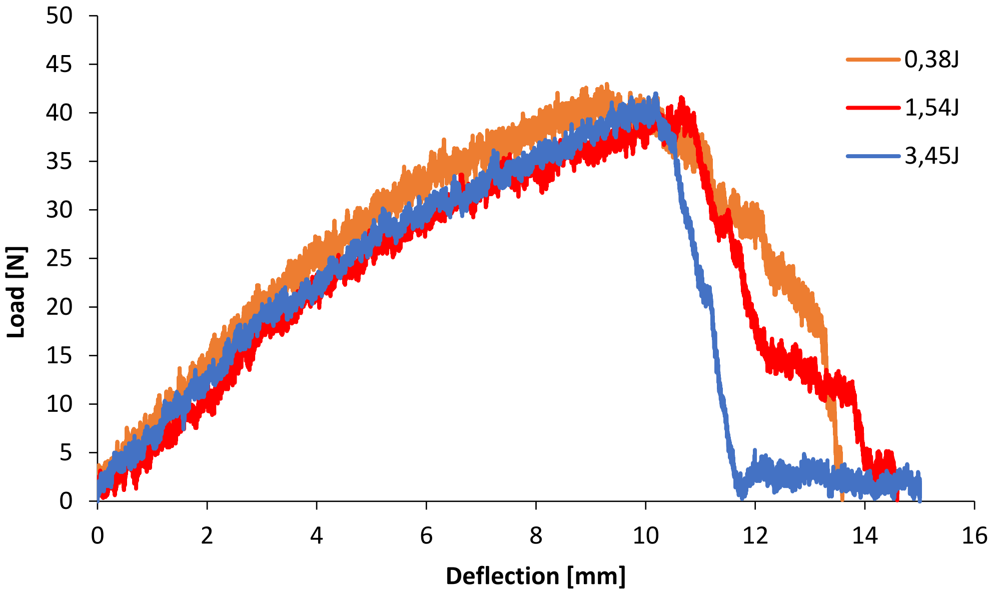

Figure S11. Load – Deflection Curves for aged PFA samples for three impact energies.

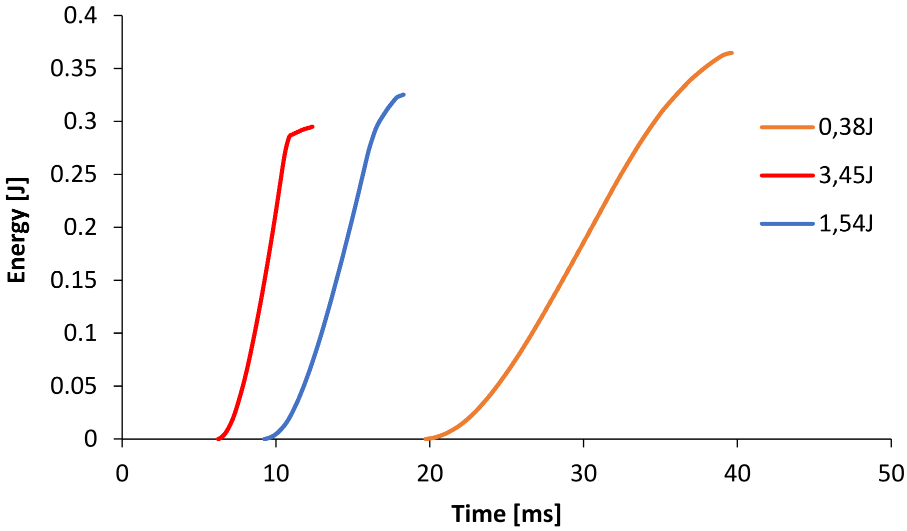

Figure S12. Energy - Time curves for aged PFA samples for three impact energies.

References

1. Andreasson,E.; Mehmood, N.; Kao-Walter, S. Trouser tear tests of two thin polymer films, Proceedings of the 13th International Conference on Fracture, June 16–21, Beijing, China, Pub International Congress on Fracture (ICF) 2013, 6-12
2. Basu, S. Determining the Critical Tearing Energy of Thin Polymer Films Using a UTM, Agilent Technologies, http://cp.literature.agilent.com/litweb/pdf/5991-0194EN.pdf, 2012
3. Gdoutos,E.E.; Schubel P.M.; Daniel, I.M., Determination of Critical Tearing Energy of Tire Rubber, Strain, 2004, 40, 119–125, 10.1111/j.1475-1305.2004.00139.x
